# Draft Genome Sequence of a “*Candidatus* Phytoplasma asteris”-Related Strain (Aster Yellows, Subgroup 16SrI-B) from South Africa

**DOI:** 10.1128/MRA.00148-19

**Published:** 2019-04-25

**Authors:** Beatrix Coetzee, Nicoleen Douglas-Smit, Hans J. Maree, Johan T. Burger, Kerstin Krüger, Gerhard Pietersen

**Affiliations:** a Department of Genetics, Stellenbosch University, Stellenbosch, South Africa; b Department of Zoology and Entomology, University of Pretoria, Pretoria, South Africa; c Citrus Research International, Stellenbosch, South Africa; d Department of Biochemistry, Genetics and Microbiology, University of Pretoria, Pretoria, South Africa; Indiana University, Bloomington

## Abstract

Here, we report the draft genome sequence of a phytoplasma discovered in grapevine. The genome size is 600,116 nucleotides (nt), with 597 predicted open reading frames. It is most similar to a maize bushy stunt phytoplasma of group 16SrI-B (aster yellows). The possible presence of a 3,833-nt plasmid was also noted.

## Abstract (precis)

Here, we report the draft genome sequence of a phytoplasma discovered in grapevine. The genome size is 600,116 nucleotides (nt), with 597 predicted open reading frames.

## ANNOUNCEMENT

Aster yellows phytoplasma of the 16SrI-B group (1, 2) was first reported in South Africa associated with grapevine yellows disease (3), and the leafhopper *Mgenia fuscovaria* was identified as the vector (4).

Healthy *Catharanthus roseus* plants were placed in a phytoplasma-infected Vitis vinifera cv. Colombar vineyard in Vredendal, South Africa, for natural transmission. After 17 weeks, samples were collected from *C. roseus*, diseased Vitis vinifera, and *M. fuscovaria* leafhoppers captured in the same vineyard. DNA was extracted from each using a cetyltrimethylammonium bromide (CTAB) method (5). Next-generation sequencing libraries were prepared with TruSeq v.3 and sequenced on the Illumina HiScanSQ platform (Agricultural Research Council, Pretoria, South Africa) to produce paired-end 2 × 100-nucleotide (nt) reads.

Sequencing of the *C. roseus* sample resulted in 15,270,318 read pairs. Read pairs were merged with PEAR v.0.9.8 (6). Quality trimming was performed on merged reads with Trimmomatic v.0.36 (7). Adaptors were removed (ILLUMINACLIP:3:30:10 parameter), 3′ and 5′ nucleotides were removed if they had a Phred quality score below 20 (LEADING:20 and TRAILING:20 parameters), and only reads with a minimum length of 20 nt were retained (MINLEN:20 parameter).

Quality-trimmed reads were aligned to the *C. roseus* genome (GenBank assembly accession number GCA_000949345) with Bowtie 2 v.2.2.8 (8). Retained unmapped reads were assembled into contigs with SPAdes v.3.13.0 (9), including the reference sequences of maize bushy stunt (GenBank accession number CP015149) and onion yellows (GenBank accession number AP006628) (the complete genome sequences of group 16SrI-B), to guide the assembly (with the SPAdes “untrusted contigs” parameter). Assembled contigs were compared to the NCBI nucleotide database with BLAST v.2.4.0 (10). Only contigs with hits to phytoplasmas were retained. The unmapped reads were again assembled in SPAdes, using these contigs as reference sequences. This process was repeated 10 times, each time retaining only contigs with BLAST hits to phytoplasmas. This yielded two contigs of 600,116 nt (28.4% GC content) and 3,833 nt (25.7% GC content) in length, representing the phytoplasma genome and a putative plasmid, respectively.

Using Prokka v.1.12 (11), 561 protein-coding sequences, 32 tRNAs, and four rRNAs were identified in the genome. Two 16S rRNA-encoding genes were identified, and their restriction digestion patterns that were generated with iPhyClassifier (12) confirmed they were in the 16SrI-B group.

This phytoplasma most closely resembles maize bushy stunt, based on genome organization and sequence identity. Using a minimum amino acid identity of 95% over at least 95% of the length, 393 (70%) of the 561 coding sequences aligned to maize bushy stunt.

The putative plasmid has five coding sequences, including a replication-associated protein with a geminivirus replication catalytic domain AL1 and a single-stranded DNA binding protein. Both these proteins share the highest amino acid identity with proteins from the rice orange leaf phytoplasma plasmid (GenBank accession numbers ATL14544 and ATL14548, with 78% and 96% shared amino acid identity, respectively).

Reads from the *V. vinifera* and *M. fuscovaria* samples were treated in the same manner and aligned to the generated phytoplasma genome and putative plasmid contigs with Bowtie 2. The *V. vinifera* data set covers 58% of the genome and 100% of the plasmid with at least one read. The *M. fuscovaria* data set covers 94.7% of the genome and 100% of the plasmid. This is good evidence that the *V. vinifera* and *M. fuscovaria* data sets contain the same phytoplasma strain (both the genome and putative plasmid) as the *C. roseus* data set.

### Data availability.

Sequencing data are available at NCBI under BioProject number PRJNA522055. Assembled genome and plasmid sequences from the *C. roseus* data are available at NCBI GenBank under accession numbers CP035949 and CP035950, respectively.
